# Mental Health, Sport, and Positive Youth Development in Prison Systems: How Can We Move Research and Practice Forward?

**DOI:** 10.3389/fpsyg.2021.598766

**Published:** 2021-03-08

**Authors:** Ana Rita Martins, Stewart Vella, Fernando Santos

**Affiliations:** ^1^Centro Hospitalar de Vila Nova de Gaia, Vila Nova de Gaia, Portugal; ^2^Faculty of Social Sciences, University of Wollongong, Wollongong, NSW, Australia; ^3^Escola Superior de Educação, Instituto Politécnico do Porto, Porto, Portugal; ^4^inEd, Centre for Educational Research and Innovation, Porto, Portugal

**Keywords:** sport, life skills, youth, challenges, social justice

## Introduction

The purpose of this opinion article is to provide insights on the need to foster mental health and Positive Youth Development (PYD) through sport within the prison system as well as identify a set of practical implications and future research directions relevant to this line of inquiry and contextualized to the Portuguese context. This is predicated on the assumption that society is characterized by a complex reality where youth ranging between 15 and 18 years old face multiple social challenges such as the need to succeed in a highly competitive environment, cope with stress, deal with a global pandemic and subsequently flourish now and in the future as adults (Lambert et al., 2020). Further, many youth are currently incarcerated, serving sentences and need support to become active contributors to society in the future (Barnert et al., 2015). For example, in the United States of America approximately more than 30.000 youth are currently incarcerated (Kaeble et al., 2015). Considering that many young people are incarcerated across the globe, there is the need to have in mind the long-term detriments associated with incarceration during adolescence such as mental health disorders and the low reintegration rate. In many cases, young people experience problems with reintegration into society after their release from prison, for instance in terms of obtaining housing and engaging in school (Anthony et al., 2009). Reintegration in society occurs *when youth return to their communities and apply skills that enable them to establish meaningful relationships with family and peers, contribute to their communities, obtain education and housing, as well as avoid deviant behaviors such as substance abuse and law violations* (Anthony et al., 2009). Statistics across contexts also show that more than 50% of incarcerated youth develop mental health disorders that create barriers for a successful reintegration in society (Cocozza and Skowyra, 2000; Walmsley, 2016). Sport has been considered a valuable platform within preparation for release that may foster increased attitudes, thinking and behavior that ultimately leads to a successful reintegration in society (Meek and Lewis, 2013).

Therefore, this opinion article provides insights into creating and delivering sport-based programs that aim to foster mental health by using the philosophical underpinnings of PYD. We use the Portuguese case as an example to drive future research and practice within the prison system as the authors have been working delivering interventions in this context.

## Mental Health, Sport, and Positive Youth Development in Prison Systems

Mental health is considered crucial for incarcerated youth because it influences the way they (a) view life and themselves, (b) respect others and the environment, and (c) look positively to the future and integrate society (Keyes, 2002; Tamminen et al., 2020). More than “fixing developmental problems,” currently mental health promotion focuses on building assets, especially important for youth inserted in the prison system (Keyes, 2002; World Health Organization, 2013). Mental health can be defined as a state of well-being whereas youth can overcome social challenges, be confident and contribute to society in a positive manner (Keyes, 2002).

PYD has been integrated in policy and programming to help youth develop skills needed strive in society and overcome existent challenges (Benson et al., 2006; Holt et al., 2016). Therefore, PYD is an asset-based approach that acknowledges the need to facilitate a successful transition to adult life by teaching youth a range of life skills such as leadership and perseverance. Life skills could be viewed as desired PYD outcomes that will support long-term mental health across vulnerable populations such as incarcerated youth. Life skills are learned within an environment (e.g., prison) and applied to one or more life domains (e.g., life outside prison). Concurrently, life skills transfer reflects the processes that lead to the application of one or more life skills (e.g., emotional control) in multiple life domains (Pierce et al., 2017; Bean et al., 2018). Life skills transfer may be considered crucial, so individuals are able to reintegrate society after serving their sentences. Learning life skills may help youth navigate through the challenges of reintegrating society. As positioned by Parker et al. (2014), sport can have an important role to play in achieving this objective:

At a practical level, what such interventions also provide is an opportunity for sport to empower young people to think positively about life, to develop coherent self-advocacy, to interact with multi-agency support and, where appropriate, to re-establish familial connections and relationships. All of which is designed to enable the participants to (re) gain a sense of active citizenship, to access a better quality of life and to give “voice” to those who, for one reason or another, may have never before been heard (p. 393).

Sport-based programs have been shown to be effective in facilitating mental health benefits (Vella et al., 2014, 2020a,b) and reintegration in society (Parker et al., 2014). Most sport-based programs that aim to foster mental health have alluded to a range of benefits such as increased physical, social, and psychological skills (Parker et al., 2014; Gallant et al., 2015; Walmsley, 2016; Chekround et al., 2018). Such programs “…help to empower participants and build their self-confidence; and enforces discipline and boundaries.” (Draper et al., 2013, p. 528) and have considered a crucial inclusion strategy. Thus, sport have been considered as valuable platforms to deliver programs that aim to develop skills useful for life in society (i.e., reintegration), including resilience and mental health literacy (Vella et al., 2020b). Some mental health programs use sport based on the assumption this context inherently led to physical, social, and psychological benefits which may not be the case. Although sport may be provided to incarcerated youth (Gallant et al., 2015), mental health promotion through may be more complex and may require deliberate efforts to teach life skills. Biddle (2016) raises the need to reflect on sport as a resource that may have a positive or negative impact on youth: “It is often believed that physical activity, such as sport, can boost self-esteem. However, the nature of participation will affect whether self-esteem is elevated or even decreased” (p. 177). Therefore, we urge researchers and practitioners, especially in the Portuguese context, to consider several important principles. Robust programs that aim to increase mental health and wellbeing could benefit from the addition of life skills as desired PYD outcomes to enable and support long-term mental health and reintegration in society. Such approach may help professionals' better control which life skills are taught through sport and reconcile mental health with PYD programming. Sport-based programs could be leveraged to teach life skills to youth, increase their overall mental health, as well as facilitate reintegration in society and life skills transfer.

While there exists a number of effective sport-based interventions to promote the mental health and wellbeing of people in prison, these programs lack solid theoretical foundations (Woods et al., 2017). We propose that the addition of a PYD lens would be completely compatible with the goals and outcomes of existing interventions, and could solve some of the problems that have been articulated in the literature. For example, sports-based interventions for young people in prison have consistently shown outcomes that include the acquisition of life skills and better reintegration outcomes (e.g., Meek and Lewis, 2013; Parker et al., 2014). However, a systematic review of studies in this area has concluded that there is currently a major gap in understand “what works, how and why” (Woods et al., 2017, p. 60), including an understanding of the psychological mechanisms through which sports-based interventions work. Such information is important in being able to implement effective programs. PYD can provide a robust theoretical basis for interventions which align with the key outcomes of those that currently exist (e.g., life skills). For example, through the Developmental Theory of PYD (Jelicic et al., 2007), clear mechanisms and processes of change are articulated—a major gap among current interventions (Woods et al., 2017). Further, through conceptualisations of PYD such as Benson et al. (2006) framework of developmental assets, a robust list of developmental assets is articulated for inclusion as key outcomes of sports-based interventions. These developmental assets refer to features crucial for a successful developmental process such as opportunities provided by family and school for adolescents to flourish. Such conceptual underpinnings could add a greater intent and purposefulness to the outcomes of interventions for people in prison. Benson et al. (2006) framework of developmental assets aligns with the developmental needs of incarcerated youth due to the focus placed on forging positive relationships with family and community that increase one's well-being. Concurrently, emphasis is placed on developing skills such as respecting rules and the needs of others, as well as empathy. These developmental assets, when embedded within programming, may have an important contribution to foster reintegration in society through the means of sport. Finally, through the Model of PYD through Sport (Holt et al., 2017), PYD through sport can offer models for the acquisition of life skills through multiple pathways such as implicit and explicit learning pathways that can help practitioners to implement effective programs within the prison system. For example, sports-based interventions with young people in prison have been “wrapped around” other programs designed to explicitly teach life skills such as life-skills mentoring (Parker et al., 2014) and explicit group activities like team skills building (Meek and Lewis, 2013). Given that sport has been advocated as a vehicle to engage hard-to-engage prisoners, using sport to achieve the outcomes of other explicitly designed programs could be beneficial. The use of a model such as the Model of PYD through Sport (Holt et al., 2017) can help practitioners plan for sport programs to achieve these outcomes through explicit teaching and implicit teaching pathways.

## Practical Implications and Future Research Directions

From a practical standpoint, there are specific populations, such as incarcerated youth, that warrant attention and carefully designed intervention programs that may help them become active contributors to society instead of “problems to be managed” and “lost causes” (Lerner et al., 2005). From a research perspective to our knowledge, no studies in the Portuguese context have used these theoretical foundations (i.e., PYD and mental health) combined to develop and assess intervention programs targeting incarcerated youth. Thus, we aim to prompt reflection about the need to understand how to use the engaging nature of sport to direct programming toward incarcerated youth.

There has been an increased concern about fostering intervention programs for incarcerated youth due to the low reintegration rates and increased recidivism (Anthony et al., 2009; Barnert et al., 2015; Sfendla et al., 2018). However, sport is not always explicitly or deliberately used to foster PYD and, in turn, foster mental health. This is cause for concern. Within the Portuguese prison system sport-based programs take a competitive approach whereas participating in championships become explicit goals and mental health promotion considered an inherent outcome. Despite the low reintegration rates and increased recidivism, sport is mainly positioned as an activity to contain deviant behaviors as a deficit-reduction approach still derives programming (General Board of Reinsertion Prison Services, 2020). However, such an approach may not be sufficient. We are not stating mental health promotion is not a concern within the prison system. Instead, we suggest that sport could be strategically used to attain better mental health outcomes.

The lack of a mental health focus within sport programming might be better understood by considering how incarcerated youth and prison time have been viewed over time (Keyes, 2002). Looking back, prison's objectives, for the most part, have focused on serving a sentence. Behan (2014) highlighted that mandated authoritarian rehabilitative programmes are problematic when determining change and authentic transformation … mandated rehabilitative programmes can lead to the appearance of, rather than real change, there may be positive elements within rehabilitative programmes that recognize and try to heal the damage that criminal activities have done to prisoners themselves and their fellow citizens (p.29).

Sport programming in prison may need to focus on education and seek for youth's strengths and qualities and help them become active contributors to society. An action contribution to society involves youth's ability to “…make connections across family, school, community, and program life” (Jacobs et al., 2017, p.31) which starts in prison and hopefully continues throughout the life span. Previous research has alluded to sport's potential in developing leadership, empathy, and perseverance which are life skills crucial for life in society (Lee and Martinek, 2013; Hemphill and Richards, 2016). However, a deficit-based and punishment-based approaches are often used to transform offenders into active contributors to society (Behan, 2014) which has limited impact. The rate of effective reintegration into society is still low (Walmsley, 2016). This an important outcome to measure the impact of intervention programs and an indicator that may drive researchers to further understand how sport may be used to foster life skills transfer (Vella et al., 2020b).

We should have in mind serving prison time does not mean youth are prepared to use the time they have outside prison in a meaningful way and not reoffend which highlights the need for asset-based approaches. PYD may serve as a useful framework to infuse into prison programming that may help keep the focus on looking at young offenders as resources to be developed and on teaching life skills. For example, programmers could include opportunities for youth to establish connections in and outside sport and ultimately become socially responsible for teaching life skills to others. This may require that professionals working in prisons (e.g., guards, teachers, nurses) receive training on PYD and life skills and create a sense of congruence across different types of interventions targeting mental health (e.g., psychotherapeutic interventions, sport-based interventions). A well-trained a multidisciplinary team may help achieve this objective.

Youth's psychosocial skills, life histories, and experiences should not be considered secondary and non-important variables for physical activity and sport programming within prisons. There might be the need to involve youth, with regards to self-realization and self-knowledge, and contribute to the development of youth's strengths from the moment incarceration begins. We should have in mind that sport is not the context to achieve this, but one context.

For better sport programming to be possible across contexts, there is the need for researchers to move into unexplored fields and develop a comprehensive understanding about how to infuse PYD, mental health and sport into prison programming. A Realist Evaluation Framework (Pawson, 2013) may help researchers understand the nature of “what works, how and why” and the underlying mechanisms behind connecting PYD, mental health and sport into prison programming. A Realist Evaluation Framework (Pawson, 2013) aims to develop change as part of a cycle that includes “Context-Mechanisms-Outcomes.” These three components may enable researchers to map the context, mechanisms, and outcomes associated to PYD programming based on the nature of the context. Sport-based programs in prison may be effective in terms of stimulating life skills and fostering mental health (i.e., outcomes) only if they initiate and provide opportunities for inmates (i.e., mechanisms) considering social and cultural conditions (i.e., context). The bulk of research (Woods et al., 2017) has focused on the outcomes derived from interventions conducted within the prison system. However, research in a variety of socio-cultural contexts that ties context and mechanisms to PYD outcomes is needed.

In the case of Portugal, we were not able to find any study that connected sport, mental health, and the prison system. There may be the need to investigate how sport are indeed used to foster mental health and what outcomes are attained. We reinforce the notion that sport can be used as engaging platforms to teach young people the psychological and behavioral skills that underpin long term mental health, alongside clinical interventions, to become better equipped for life outside prison. Future studies may also attempt to study the long-term benefits associated to sport programming in prison that includes a PYD focus. Thus, three research questions may need to be posed: how can we foster PYD and mental health through sport? what are the short and long-term outcomes of such interventions? and how should they be implemented to maximize effectiveness and congruence? Answering these questions is crucial and will influence the next years of programming as mental health promotion and PYD are becoming urgent in a world whereas social issues and a global pandemic are cause for concern. More so, sport should be carefully used as it can foster both positive and negative outcomes (Vella et al., 2020a).

## Final Thoughts

To operationalize sport-based programs with an explicit PYD and mental health focus within prison systems, there might be the need to consider the importance of a multidisciplinary team (e.g., specialist nurses, kinesiology professionals, psychologists, social workers) that create a greater likelihood of reintegration into society through explicit life skill transfer processes and strategies and a sense of congruence. The ultimate and most complex objective is to make youth active contributors to society that influence people around them (e.g., other inmates, family, friends—by this order). Thus, there is the need to develop research designs that involve process and outcome evaluations of current programming and the development and implementation of novel programs that infuse a PYD and life skill focus, considering that transfer is the key objective of any prison system, specifically for youth. Real world change and meaningful research are crucial to help youth strive in life as incarcerated youth deserve and need attention. We hope this exciting line of inquiry urges others to make a difference in Portugal and elsewhere and reflect how to “properly structure” (General Board of Reinsertion Prison Services, 2020) physical activity and sport across prison systems.

## Author Contributions

AM conceptualized the manuscript and writing and editing. SV and FS writing and editing. All authors contributed to the article and approved the submitted version.

## Conflict of Interest

The authors declare that the research was conducted in the absence of any commercial or financial relationships that could be construed as a potential conflict of interest.
